# Clinicopathological Significance and Prognostic Value of DNA Methyltransferase 1, 3a, and 3b Expressions in Sporadic Epithelial Ovarian Cancer

**DOI:** 10.1371/journal.pone.0040024

**Published:** 2012-06-29

**Authors:** Xuefeng Bai, Zhiguo Song, Yingzi Fu, Zhaojin Yu, Lin Zhao, Haishan Zhao, Weifan Yao, Desheng Huang, Xiaoyi Mi, Enhua Wang, Zhihong Zheng, Minjie Wei

**Affiliations:** 1 Department of Pharmacology, China Medical University, Shenyang, Liaoning, China; 2 Department of Mathematics, College of Basic Medical Sciences, China Medical University, Shenyang, Liaoning, China; 3 Department of Pathology, College of Basic Medical Sciences, China Medical University, Shenyang, Liaoning, China; 4 Institute of Pathophysiology, China Medical University, Shenyang, Liaoning, China; Instituto Butantan, Laboratório Especial de Toxinologia Aplicada, Brazil

## Abstract

Altered DNA methylation of tumor suppressor gene promoters plays a role in human carcinogenesis and DNA methyltransferases (DNMTs) are responsible for it. This study aimed to determine aberrant expression of DNMT1, DNMT3a, and DNMT3b in benign and malignant ovarian tumor tissues for their association with clinicopathological significance and prognostic value. A total of 142 ovarian cancers and 44 benign ovarian tumors were recruited for immunohistochemical analysis of their expression. The data showed that expression of DNMT1, DNMT3a, and DNMT3b was observed in 76 (53.5%), 92 (64.8%) and 79 (55.6%) of 142 cases of ovarian cancer tissues, respectively. Of the serious tumors, DNMT3a protein expression was significantly higher than that in benign tumor samples (*P* = 0.001); DNMT3b was marginally significant down regulated in ovarian cancers compared to that of the benign tumors (*P* = 0.054); DNMT1 expression has no statistical difference between ovarian cancers and benign tumor tissues (*P* = 0.837). Of the mucious tumors, the expression of DNMT3a, DNMT3b, and DNMT1 was not different between malignant and benign tumors. Moreover, DNMT1 expression was associated with DNMT3b expression (*P* = 0.020, r = 0.195). DNMT1 expression was associated with age of the patients, menopause status, and tumor localization, while DNMT3a expression was associated with histological types and serum CA125 levels and DNMT3b expression was associated with lymph node metastasis. In addition, patients with DNMT1 or DNMT3b expression had a trend of better survival than those with negative expression. Co-expression of DNMT1 and DNMT3b was significantly associated with better overall survival (*P* = 0.014). The data from this study provided the first evidence for differential expression of DNMTs proteins in ovarian cancer tissues and their associations with clinicopathological and survival data in sporadic ovarian cancer patients.

## Introduction

Epigenetic alteration of the genomic DNA refers to functionally relevant modifications of the genome that affects gene expression but do not involve a change in the nucleotide sequence, which plays an important role in human carcinogenesis. Such modifications include DNA methylation, chromatin remodeling and histone variants, and the epigenetic function of non-coding RNA [1]. Among these, DNA methylation is a covalent modification of DNA that plays an important role in setting gene expression programs during development [2]. Nevertheless, abnormal DNA methylation does also play an important role in human cancer development, and most cancer cells show a global hypomethylation of the genome that induces abnormal expression of genes but a local hypermethylation that silences tumor suppressor genes [1], [3]. In mammals, DNA methylation is established and maintained mainly by three DNA methyltransferases (DNMTs), namely DNMT1, DNMT3a, and DNMT3b. The preferred target of DNMT1 is hemi-methylated DNA [4], [5] and this protein functions as a “maintenance” methyltransferase and the primary enzyme responsible for copying methylation patterns after DNA replication. It localizes to replication foci and interacts with proliferation cell nuclear antigen (PCNA) [6]. In contrast, DNMT3a and DNMT3b are essential for early embryonic development and responsible for de novo methylation [7]. Overexpression of these three DNMTs has been reported in various malignancies and associated with poor survival of different cancers, including lung, liver, and cervical cancers and lymphomas [8], [9], [10], [11], [12], [13], [14], [15], [16], [17], [18]. Unlike cancer-associated gene mutations, amplifications, and deletions, DNA methylation is potentially reversible. Thus, DNMTs have been investigated as a target for re-expression of tumor suppressor genes and reversal of malignant phenotypes in different malignancies [19]. Most recently, antisense oligonucleotides targeting DNMT genes, for example MG98, appear to be effective in preclinical studies and has now entered into clinical phase I and phase II studies [20], [21].

Epithelial ovarian cancer is the sixth leading cancer in women worldwide and the second most common gynecologic cancer, accounting for approximately 4% of all female cancers [22], [23]. Due to lacking early warning signs and effective screening tools, majority of patients present with late stages of the disease [24]. Epithelial ovarian cancer is the most lethal gynecologic malignancy and the five-year survival rate is below 25% for patients diagnosed with stage III - IV diseases [25], [26]. Similar to other malignancies, aberrant DNA methylation on CpG islands is also an important mechanism for ovarian cancer development. Preclinical studies indicated that de-methylation agents were able to reverse resistance of ovarian cancer cells to platinum [27], [28], leading to using DNMT inhibitors in clinical trials of ovarian cancers [29], [30], [31], [32]. However, there are no data available on study of DNMT proteins in ovarian cancer tissues, although two previous studies have reported the expression of DNMT mRNA in ovarian cancer cell lines and small tissue samples [33], [34]. In this study, we collected 142 cases of epithelial ovarian carcinoma samples and 44 cases of benign ovarian tumors for detection of DNMT1, DNMT3a, and DNMT3b protein expressions in order to determine the role of DNMT proteins in ovarian cancer and clinical significance.

## Results

### Patient Characteristics

In this study, we recruited tissue samples from 186 ovarian tumor samples for evaluation of DNMTs protein expression. The clinicopathological data from the patients are shown in Table 1. Briefly, the mean age of the patients at surgery was 53 years (ranging from 20 to 74 years). 27 (23.3%) patients had lymph node-metastasized disease at the time of surgery and 110 (77.5%) patients had serous carcinoma as the main histological diagnosis, followed by mucinous carcinoma (8.5%), clear cell carcinoma (∼5.6%), and undifferentiated carcinoma (∼5.6%). The serum levels of CA125, CA199, and CEA were elevated before surgery but none of the patients received any neo-adjuvant chemotherapy. Follow-up data were available for 85 patients. The mean and median overall survivals (OS) were 56.1 and 41.0 months, respectively, with a 95% confidence interval of 45.3 to 66.9 and 33.2 to 48.8 months. The mean and median disease-free survivals (DFS) were 46.6 and 26.0 months, respectively, with a 95% confidence interval of 36.4 to 56.9 and 14.6 to 37.4 months. In the 44 benign tumors, 31 were serous and 13 mucinous.

**Table 1:** Patient characteristics.

Feature	Categories	Number	%
**Age, years**	≤53	79	57.2
	>53	59	42.8
	unknown	4	–
**Menopause state**	pre-menopause	45	34.6
	post-menopause	85	65.4
	unknown	12	–
**Histological type**	serous	110	77.5
	mucinous	12	8.5
	clear cell	8	5.6
	transitional	2	1.4
	endometrioid	2	1.4
	undifferentiated	8	5.6
**Tumor size, cm**	≤5	15	12.5
	5∼10	53	44.2
	>10	51	43.3
	unknown	23	–
**FIGO stage**	I∼II	31	24.4
	III∼IV	96	75.6
	unknown	15	–
**Nodes metastasis**	no	89	76.7
	yes	27	23.3
	unknown	26	–
**Location of tumor**	single side	56	43.4
	both sides	73	56.6
	unknown	13	–
**CA125, U/ml**	0∼35	10	10.1
	35∼500	40	40.4
	500∼1000	32	32.3
	>1000	17	17.2
	unknown	43	–
**CA199, U/ml**	0∼37	67	76.1
	37∼100	10	11.4
	>100	11	12.5
	unknown	54	–
**CEA, ng/ml**	0∼5	72	91.1
	>5	7	8.9
	unknown	63	–
**Chemotherapy**	platinum-based	111	94.9
	nonplatinum	3	2.6
	No chemotherapy	3	2.6
	unknown	25	–

### Differential Expression of DNMT Proteins in Malignant and Benign Ovarian Tumors

**Figure 1:**
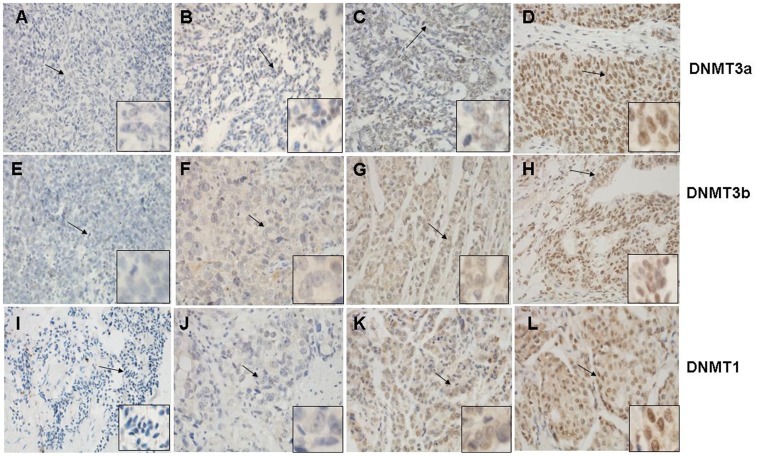
Immunohistochemical staining of DNMT3a, DNMT3b, and DNMT1 proteins in ovarian cancer tissues Representative examples of negative (A, E, I), weakly positive (B, F, J), moderately positive(C, G, K), and strong positive (D, H, L) immunostaining for DNMT3a, DNMT3b, and DNMT1 expression are shown, respectively; Arrows indicate the field enlarged. Magnification: ×400; enlarged sites: ×1000.

**Figure 2:**
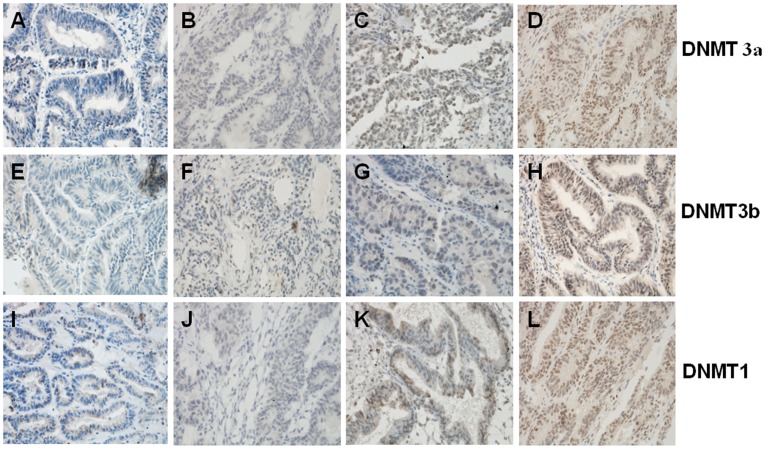
Immunohistochemical staining of DNMT3a, DNMT3b, and DNMT1 proteins in benign ovarian tumors Representative examples of negative (A, E, I), weakly positive (B, F, J), moderately positive(C, G, K), and strong positive (D, H, L) immunostaining for DNMT3a, DNMT3b, and DNMT1 expression are shown, respectively. Magnification: ×400.

Differential expression of DNMT proteins in malignant and benign ovarian tumors according to histological type is summarized in Table 2. In particular, the expression of DNMT3a protein was significantly higher than that of benign tumor tissues (Mann-Whitney U-test test, *P* = 0.001; Table 2), but the difference was not observed in mucious tumors (Mann-Whitney U-test test, *P* = 0.813; Table 2). While DNMT3b protein was detected in 64 (58.2%) of the 110 serous cancer cases and 22 (71.0%) of the 31 serous benign tumors, indicating that DNMT3b expression in ovarian cancers was lower but not significantly than that of benign tumors (Mann-Whitney U-test test, *P* = 0.054; Table 2), but the difference was not observed in mucious tumors (Mann-Whitney U-test test, *P* = 0.536; Table 2). In contrast, the expression of DNMT1 protein was not significantly different between in ovarian cancer and benign tumor samples either in serious or in mucious tumors (Mann-Whitney U-test test, serous: *P* = 0.837; mucious: *P* = 0.315 Table 2). Representative expression patterns of immunohistochemical staining of DNMTs in ovarian cancer and benign tumor tissues were shown in Figure 1 and Figure 2 respectively. Next, we associated expression of these three DNMT proteins in the ovarian cancer tissues by using Spearman’s rank correlation test. The data showed that expression of DNMT1 was significantly associated with DNMT3b (r = 0.195, *P* = 0.020; Table 3), but not with DNMT3a protein (r = 0.130, *P* = 0.122; Table 3) or between the DNMT3a and DNMT3b proteins (r = 0.152, *P* = 0.071; Table 3).

**Table 2:** DNMTs expression in malignant and benign ovarian tumors.

			−	+	++	+++		
		n	n (%)	n (%)	n (%)	n (%)	PR^ a^, %	*P-*value b
**serous**	**DNMT1**							
	Malignant	110	51 (46.4)	46 (42.6)	7 (5.5)	6 (5.5)	53.6	0.837
	Benign	31	17 (54.8)	4 (12.9)	6 (19.4)	4 (12.9)	45.2	
	**DNMT3a**							
	Malignant	110	34 (30.9)	31 (28.2)	25 (22.7)	20 (18.2)	69.1	0.001
	Benign	31	22 (71.0)	5 (16.1)	1 (32.2)	3 (9.7)	29.0	
	**DNMT3b**							
	Malignant	110	46 (41.8)	36 (32.7)	18 (16.4)	10 (9.1)	58.2	0.054
	Benign	31	9 (29.0)	8 (25.8)	5 (16.1)	9 (29.0)	71.0	
**mucious**	**DNMT1**							
	Malignant	12	5 (41.7)	6 (50.0)	0 (0)	1 (8.3)	58.3	0.315
	Benign	13	5 (38.5)	2 (15.3)	3 (23.1)	3 (23.1)	62.5	
	**DNMT3a**							
	Malignant	12	8 (66.7)	3 (25.0)	1 (8.3)	0 (0)	33.3	0.813
	Benign	13	8 (61.5)	3 (23.1)	2 (15.4)	0 (0)	38.5	
	**DNMT3b**							
	Malignant	12	5 (41.7)	5 (41.7)	0 (0)	2 (16.6)	58.3	0.536
	Benign	13	7 (53.8)	4 (30.8)	2 (15.4)	0 (0)	46.2	

− negative; + weak; ++ moderate; +++ strong staining; ^a^PR, positive rate.

b *P*-value obtained from Mann-Whitney U-test test.

**Table 3:** Correlations between DNMTs expression in ovarian cancers.

		DNMT3a		DNMT3b	
Features	n	r^a^	*P*–value^b^	r^a^	*P*–value^b^
DNMT1	142	0.130	0.122	0.195	0.020
DNMT3a	142			0.152	0.071

a Spearman’s coefficient of correlation;

b *P*-value obtained from Spearman’s correlation.

### Association between Expression of DNMT Proteins and Clinicopathological Parameters

After detected expression of these three DNMT proteins using immunohistochemistry, we associated their expressions with clinicopathological data from the patients. Our data showed that DNMT1 expression was significantly associated with age and menopause state of the patients and with tumor localization. Expression of DNMT1 protein in the older (>53 years) or postmenopausal patients was higher than that of younger patients (Pearson Chi-Square test, *P* = 0.028; Table 4) or premenopausal patients (Pearson Chi-Square test, *P*<0.000; Table 4). Moreover, expression of DNMT1 protein was higher in the patients whose tumor occurred in single side than that of patients whose tumor occurred in both sides (Pearson Chi-Square test, *P* = 0.010; Table 4). However, there were no significant associations present between DNMT1 immunoreactivity and tumor size, lymph node metastasis, clinical stage, histological type, CA125, or CA199.

**Table 4:** Correlation between DNMTs expression and clinicopathological features of sporadic ovarian cancer patients.

Features		n	DNMT1 n (%)^a^	DNMT3a n (%)	DNMT3b n (%)	DN3a+DN3b n (%)	DN1+DN3a n (%)	DN1+DN3B *N (%)*
**Age at diagnosis**		138						
**≤ 53 (year)**		79	36(45.6)	53 (67.1)	42 (53.2)	33(41.8)	27(34.2)	23(29.1)
**>53 (year)**		59	38(64.4)	37 (62.7)	35 (59.3)	25(42.4)	25(42.4)	26(44.1)
	*P* b		0.028	0.593	0.471	0.944	0.326	0.069
**Menopause state**		130						
**Pre- Menopause**		45	15(33.3)	29 (64.4)	20(44.4)	18(40.0)	10(22.2)	8(17.8)
**Post- Menopause**		85	56(65.9)	54 (63.5)	51(60.0)	35(41.2)	39(45.9)	40(47.1)
	*P*		0.000	0.918	0.090	0.897	0.008	0.001
**Tumor size(cm)**		119						
**≤ 5.0 cm**		15	7(46.7)	8(53.3)	7(46.7)	3(20.0)	6(40.0)	4(26.7)
**5∼10 cm**		53	32(60.4)	33(62.3)	28(52.8)	19(35.8)	18(34.0)	18(34.0)
**>10 cm**		51	27(52.9)	33(64.7)	30(58.8)	26(51.0)	19(37.3)	19(37.3)
	*P*		0.571	0.727	0.665	0.066	0.890	0.746
**Nodes metastasis**		116						
**no**		89	48 (53.9)	59 (66.3)	48 (53.9)	41(46.1)	35(39.3)	31(34.8)
**yes**		27	14 (51.9)	16 (59.3)	21 (77.8)	12(44.4)	9(33.3)	12(44.4)
	*P*		0.849	0.503	0.027	0.882	0.574	0.365
**FIGO stage**		127						
**I∼II**		31	18 (58.1)	22 (71.0)	18 (58.1)	17(54.8)	12(38.7)	11(35.5)
**III∼IV**		96	51 (53.1)	63 (65.6)	53 (55.2)	39(40.6)	36(37.5)	34(35.4)
	*P*		0.631	0.583	0.781	0.166	0.904	0.995
**Histological type**		130						
**serous**		110	59 (53.6)	76 (69.1)	64 (58.2)	48(43.6)	45(40.9)	41(37.3)
**mucinous**		12	7 (58.3)	4 (33.3)	7 (58.3)	6(50.0)	5(41.7)	4(33.3)
**clear cell**		8	7 (87.5)	4 (50.0)	4 (50.0)	3(37.5)	2(25)	4(50.0)
	*P*		0.200	0.027	0.933	0.853	0.670	0.754
**Location of tumor**		129						
**single side**		56	38 (67.9)	37 (66.1)	31 (55.4)	23(41.1)	27(48.2)	27(48.2)
**both sides**		73	33 (45.2)	49 (67.1)	41 (56.2)	32(43.8)	22(30.1)	20(27.4)
	*P*		0.010	0.900	0.927	0.753	0.036	0.015
**CA125, U/ml**		89						
**35∼500**		40	25 (62.5)	22 (55.0)	21 (52.5)	14(35.0)	14(35.0)	15(37.5)
**500∼1000**		32	18 (56.2)	18 (56.2)	14 (43.8)	11(34.4)	11(34.4)	10(31.2)
**>1000**		17	7 (41.2)	15 (88.2)	9 (52.9)	8(47.1)	8(47.1)	6(35.3)
	*P*		0.332	0.044	0.723	0.638	0.638	0.917
**CA199, U/ml**		88						
**0∼37**		67	30 (44.8)	40 (59.7)	29 (43.3)	25(37.3)	19(28.4)	16(23.9)
**>37**		21	13 (61.9)	15 (71.4)	14 (66.7)	11(52.4)	9(42.9)	11(52.4)
	*P*		0.171	0.333	0.061	0.220	0.213	0.013

a Numbers in parentheses are percentage.

b *P*-value obtained from Pearson Chi-Square or Fisher’s exact test.

Furthermore, there was a significant association between DNMT3a and serum CA125 level (Pearson Chi-Square test, *P* = 0.044; Table 4). Expression of DNMT3a protein was 69.1% in serous carcinoma, which was higher than that of mucinous carcinoma (33.3%) and clear cell carcinoma (50%) (Fisher’s exact test, *P* = 0.027; Table 4). But there was no association between DNMT3a immunoreactivity and other clinical pathological parameters, as displayed in Table 4.

In addition, the data revealed that DNMT3b expression was significantly associated with lymph node metastasis of ovarian cancer (Pearson Chi-Square test, *P* = 0.027; Table 4). Although there is not statistically significant, expression of DNMT3b protein appeared to be associated with high level of serum CA199 (Pearson Chi-Square test, *P* = 0.061; Table 4).

Co-expression of DNMT1 and DNMT3a was significantly associated with menopause state (Pearson Chi-Square test, *P* = 0.008; Table 4) and location of the tumor (Pearson Chi-Square test, *P* = 0.036; Table 4), while co-expression of DNMT1 and DNMT3b was significantly associated with menopause state (Pearson Chi-Square test, *P* = 0.001; Table 4) and location of the tumor (Pearson Chi-Square test, *P* = 0.015; Table 4). Co-expression of DNMT1 and DNMT3b was also significantly associated with the serum CA199 levels (Pearson Chi-Square test, *P* = 0.013; Table 4).

### Association of DNMT Protein Expressions with Survival of the Patients

**Figure 3:**
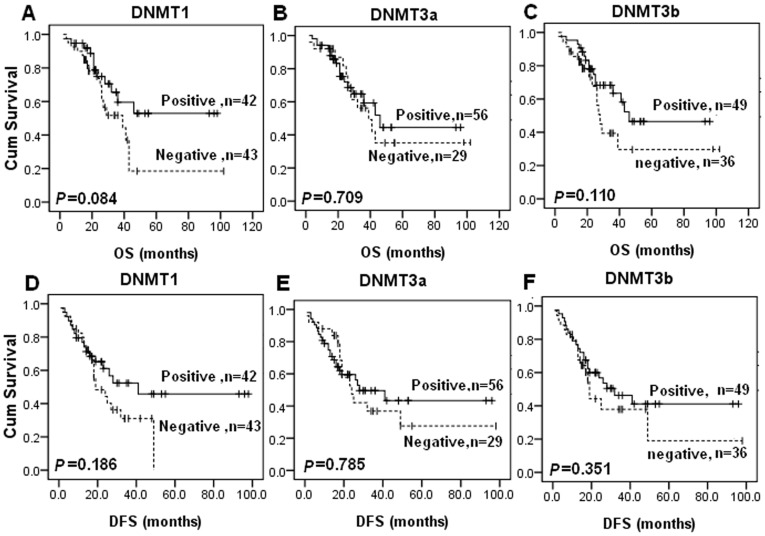
Kaplan-Meier estimates of patients with ovarian cancer stratified by DNMTs protein expression Survival curves show that although there was no statistical significance, patients with DNMT1 expression had a trend of improved overall survival (A) and disease-free survival (D) than those with negative expression, and so did DNMT3b (C and F). But there is no significant difference between DNMT3a expression and overall survival or disease-free survival (B and E). *P*-value obtained from the log-rank test.

**Figure 4:**
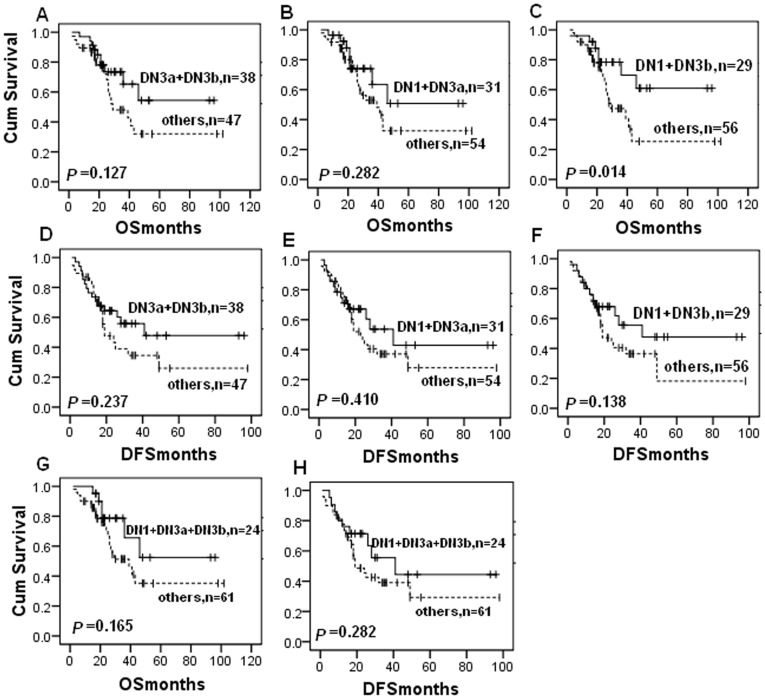
Kaplan-Meier estimates of patients with ovarian cancer stratified by co-expression of DNMTs. Survival curves show that co-expression of DNMT1 and DNMT3b was significantly associated with improved overall survival compared to negative samples (*P* = 0.014, Fig. 3E). *P*-value obtained from the log-rank test.

After that, we associated expression of DNMT protein with survival of the patients using Kaplan-Meier analysis (Figure 3). Particularly, although it is not significantly different, patients with DNMT expressions had a trend of improved survival than those with negative expression, opposite of findings from previous studies in other types of cancer [8], [13], [17], [18], [35], [36]. In our samples, DNMT1 expression marginally associated with improved OS (*P* = 0.084; Fig. 3A) and DFS (*P* = 0.186; Fig. 3D); and so did DNMT3b. Moreover, co-expression of DNMT1 and DNMT3b was significantly associated with improved overall survival compared to other samples (only DNMT1 or DNMT3b expression or both negative) (*P* = 0.014, Fig. 4C). Univariate analysis of the potential prognostic impact of clinical and histopathological parameters identified clinical stage, location of tumor, and serum CA125 level as significantly or marginally significantly associated with shorter OS and DFS (Table 5).

**Table 5:** Univariate Cox regression analysis of clinical and pathological data correlated with OS and DFS in total ovarian cancers.

Factor		Overall survival			Disease-free survival		
	n	RR a	95%CI b	*P*	RR	95%CI	*P*
**Age, years**	85	1.283	0.638∼2.583	0.485	1.330	0.724∼2.442	0.357
**>53/≤53**							
**Menopause state**	83	1.187	0.532∼2.649	0.676	1.335	0.668∼2.667	0.413
**post/pre**							
**Tumor size, cm**	70	0.277	0.102∼0.752	0.012	0.373	0.171∼0.812	0.013
**>10/≤10.0**							
**Nodes metastasis**	68	1.695	0.708∼4.055	0.236	1.114	0.483∼2.571	0.799
**yes/no**							
**Histological type**	84	0.950	0.411∼2.197	0.904	0.886	0.423∼1.853	0.748
**serousr/non-serous**							
**FIGO stage**	79	8.750	2.072∼36.951	0.003	7.446	2.279∼24.331	0.001
**III∼IV/I∼II**							
**Location of neoplasia**	78	2.019	0.931∼4.376	0.075	2.227	1.112∼4.462	0.024
**both sides/single side**							
**CA125, U/ml**	56	1.688	0.884∼3.224	0.113	2.067	1.218∼3.508	0.007
**>1000/500∼1000/35∼500**							
**CA199, U/ml**	53	0.366	0.085∼1.579	0.178	0.399	0.119∼1.342	0.138
**>37/0∼37**							
**DNMT1 stutas**	85	0.525	0.260∼1.062	0.073	0.640	0.347∼1.178	0.152
**positive/negative**							
**DNMT3a stutas**	85	0.875	0.437∼1.754	0.708	0.867	0.470∼1.601	0.649
**positive/negative**							
**DNMT3b stutas**	85	0.583	0.291∼1.167	0.128	0.786	0.425∼1.453	0.442
**positive/negative**							

a RR, relative risk;

b 95% CI, 95% confidence interval.

Subsequently, multivariate Cox regression models using clinical stage, tumor size, location of neoplasia, DNMT1 and DNMT3b co-expression revealed that only clinical stage (OS, *P* = 0.022, RR = 6.977, 95% CI: 1.322∼36.822; DFS, *P* = 0.026, RR = 4.686, 95% CI: 1.207∼18.196) remained as an independent prognostic factor (Table 6).

**Table 6:** Multivariate Cox Regression analysis of OS and DFS in ovarian cancers.

	Overall survival		Disease-free survival	
Category	RR^a^ (95% CI^b^)	*P*	RR (95% CI)	*P*
**Clinicalstage (III∼IV/I∼II)**	6.977 (1.322∼36.822)	0.022	4.686 (1.207∼18.196)	0.026
**Tumor size,cm (>10/≤10.0)**	0.566 (0.188∼1.706)	0.312	0.546 (0.226∼1.322)	0.180
**Location of neoplasia (both/single side)**	0.581 (0.195∼1.732)	0.330	0.943 (0.363∼2.452)	0.904
**DN1+DN3b (positive/negative)**	0.493 (0.170∼1.429)	0.193	0.689 (0.286∼1.659)	0.406

a RR, relative risk;

b 95% CI, 95% confidence interval.

### Subgroup Analysis of Association between DNMT Expressions and Clinical Outcome of the Patients

Further analysis was performed with regard to DNMTs expression in subsets of patients with different clinicopathological parameters, such as age, menopause state, tumor size, clinical stage, lymph node metastasis, and location of the tumor. Our data showed that expression of DNMT1 protein was associated with improved DFS in patients with larger size of tumors (DFS, *P* = 0.027; Figure S1). Expression of DNMT1 appeared to be a protective factor in patients whose tumor occurred in both sides but it is not statistically significant (OS, *P* = 0.139; Figure S1) (DFS, *P* = 0.269; Figure S1); opposite of findings from patients whose tumor occurred in single side (OS, *P* = 0.429; Figure S1) (DFS, *P* = 0.210; Figure S1). In contrast, DNMT3a protein levels failed to show any associations with patient survival (*P*>0.05, Figure S2). DNMT3b expression was associated with prolonged OS in older patients (*P* = 0.011; Figure S3), postmenopausal patients (*P* = 0.019; Figure S3), and patients whose tumor occurred in both sides (*P* = 0.022; Figure S3). Expression of DNMT3b protein also marginally associated with prolonged DFS in postmenopausal patients (*P* = 0.098; Figure S3) and patients whose tumor occurred in both sides (*P* = 0.097; Figure S3).

## Discussion

In the present study, we for the first time immunohistochemically determined the expression of DNMT1, DNMT3a, and DNMT3b proteins in benign and malignant ovarian tumor tissues. The data showed that DNMT3a expression was higher in ovarian cancer than that in benign tumors, which was consistent with the previous studies in other types of cancer [15], [37], [38]. However, although several previous studies have shown that expression levels of DNMT3b mRNA [8], [9], [39], [40] and protein [13], [40], [41] were increased in a variety of malignant tumors, our current data demonstrated a lower expression of DNMT3b in ovarian cancer tissues compared to that of benign tumor. In this study, we also found that there was no difference in expression of DNMT1 protein between ovarian benign and malignant tissues. It is agreeable to a previous study reporting that there was no difference in DNMT1 mRNA expression among normal ovarian tissue, primary ovarian cancer, and recurrence of ovarian cancer tissues [33]. Another report demonstrated that expression of DNMT1 mRNA levels in ovarian cancer HeyA8 and HeyC2 cell lines was higher than that of normal ovarian epithelial cells [34]. Our current study detected for the first time expression of DNMT proteins in ovarian cancer by compared to the benign tissues; thus further study is needed to analyze the altered expression of DNMT proteins in ovarian cancer with the comparison with normal ovarian tissues. In any events, due to the complex mechanisms responsible for regulation of DNMT expressions and functions of DNMTs in carcinogenesis, the altered expression and effects of DNMTs should be further investigated in ovarian cancer although their aberrant expressions were found to be because of methylation of their gene promoters in different cancers, such as gliomas and embryonic tissues [42], [43]. However, mutation and loss of expression of p53 protein led to overexpression of DNMT1 in leukemia, colorectal cancer and lung cancer [44]. In addition, microRNAs are also involved in regulation of DNMT expression [45], [46]. Borderline epithelial tumor was a significant and important group of epithelial tumor of the ovary. We have also collected borderline epithelial tumors but the number of borderline tumors available in our study were only 6, so we do not analyzed the borderline epithelial tumors in this study, and we will continue to collect more borderline tumors for future study.

Moreover, our current study further associated the relevance of three DNMT protein expressions with clinicopathological features from ovarian cancer patients. The data showed that DNMT1 expression was positively correlated with age of the patients, i.e., DNMT1 protein was expressed more in older patients, the data of which were consistent with that of lymphoma [13]. Moreover, DNMT1 protein was expressed more in post-menopausal patients than that in pre-menopausal patients. In addition, our data showed that expression of DNMT1 protein was relevant with the localization of the tumor. However, to date, we don’t know why these happened or the implication of these associations. In addition, we also found that expression of DNMT1 was higher in the unilateral ovarian cancer than in bilateral ovarian cancer, which may indicate the difference of the unilateral and bilateral ovarian cancer in terms of the biological characteristics, genetics, and mechanism. A previous study has reported that DNMT1 expression was associated with lymph node metastasis in pancreas cancer [17], but we did not find such an association in ovarian cancer.

In addition, our current study showed that DNMT3a expression was associated with histological type, e.g., ovarian serous carcinoma expressed higher levels of DNMT3a protein. DNMT3a expression was positively associated with serum CA125 level, while DNMT3b expression was associated with lymph node metastasis and serum CA199 level, which is novel and was not report before.

In terms of survival prediction using different DNMT expressions, previous studies showed that overexpression of DNMT1 or DNMT3b was associated with a poor prognosis in cancers of the lung [15], liver [18], [35], and pancreas [17], lymphoma [13] and other malignancies. In ovarian cancer, our current study showed a different trend of DNMT associations with prognosis, i.e., the overall survival (OS) and disease-free survival (DFS) of ovarian cancer patients were better with expression of DNMT1 and DNMT3b proteins compared to the patients without expression of DNMT1 and DNMT3b proteins. Moreover, co-expression of DNMT1 and DNMT3b proteins was also significantly associated with improved OS. Moreover, our additional results showed that DNMT3a in different subsets of patients with ovarian cancer had no effect on the survival status, although DNMT1 expression showed better OS and DFS in patients with large tumors than that in patient with smaller tumors. The similar is true for DNMT3b expression in older patients, postmenopausal patients, and patients with bilateral tumors positive. These completely different data from the current study suggest different biological characteristics of ovarian cancer from other cancers. However, future study is needed to confirm our current finding.

In a variety of cancers, DNMT1, DNMT3a, and DNMT3b were reported to be highly expressed and associated with poor prognosis. Hypermethylation of some tumor suppress gene promoters (TSG) that affect the prognosis can partially explain the poor prognosis associated with patients with DNMT overexpression [47], [48], [49], [50], although methylation of most gene promoters doesn’t have high correlation with the expression of DNMTs [35], [51]. However, it is unknown why high expression of some of these DNMTs was associated with better prognosis of ovarian cancer patients. Previous studies demonstrated that methylation of DNA CpG island in tumor tissues was not the only part of the hypermethylation of genes, that hypomethylation also occurred in some regions of genomic DNA, and that abnormal hypomethylation in human genome affected prognosis of patients with prostate cancer, liver cancer, and glioma [52], [53], [54]. Like other cancers, ovarian cancer cells also show a global hypomethylation in the genome and a local hypermethylation of tumor suppressor gene promoters [55]. Two previous studies used methylation chip as the high-throughput method to screen gene hypermethylations that may affect the prognosis of patients with ovarian cancer [56], [57] and found that a higher degree of CpG island methylation is associated with reduced patient progression-free survival (PFS). While other studies reported that promoter hypermethylation was involved in DNA damage repair genes in ovarian cancer and associated with improved prognosis of patients through increased the sensitivity to chemotherapy [58], [59]. In addition, recent studies showed that DNMT inhibitor was only able to partially reverse platinum resistance in patients with ovarian cancer [32]. Another study evaluated the activity and tolerability of a demethylation agent fazarabine (Ara-AC) in patients with ovarian cancer, and no complete or partial responses were observed in the 19 patients [29]. Taken altogether, our current study indicates that the mechanism and clinical significance of altered expression of DNMTs in ovarian cancer could be further evaluated.

## Materials and Methods

### Tissue Specimens

Tissue samples from 186 patients with ovarian tumors were recruited from the Department of Surgical Oncology and General Surgery, China Medical University-Affiliated First and Second Hospitals between 2002 and 2010. Out of 186 cases, 142 cases were histologically confirmed as ovarian epithelial carcinoma and 44 cases were benign ovarian tumors. None of our patients had any family history of cancer. The patients were surgically staged according to the current FIGO (International Federation of Gynecologists and Obstetricians classification system). Histological diagnosis was reached based on the criteria of the World Health Organization. Our study was attached to another clinical trial which we obtained informed consent from all participants. Because this trial was about DNMT inhibitor and we want to know the clinicopathological significance and prognostic value of DNMT expressions in sporadic ovarian cancer. So we like to add this study about DNMT expression. We called to each patient, explained our study, and obtained informed consent. When we called, a notary public was present and will give testimony, and we received the mobile phone short message which we required participants who consent the study to send to us. Because the time was limited and some participants were out of the hospital, so it is difficult to obtain written consent. The Institute Research Medical Ethics Committee of China Medical University discussed and approved the consent procedure. Finally, we obtained verbal consent from 186 patients.

### Immunohistochemistry

Formalin-fixed and paraffin-embedded tissue samples were cut into 4-µm thick sections and mounted onto poly-L-lysine-coated glass slides. For immunohistochemical staining, the sections were deparaffinized in xylene, rehydrated in a series of alcohol, and washed in the tap water. The sections were then cooked in 10 mM sodium citrate buffer, pH 6.0, for 10 min in an autoclave for antigen retrieval. Endogenous peroxidase activity was blocked by incubating the sections in 3% H_2_O_2_ at 37°C for 20 min. after that, the sections were blocked to avoid nonspecific binding by addition of a 10% normal goat serum at 37°C for 30 min and then incubated for 4°C overnight with the polyclonal antibody against DNMTs (DNMT1, sc-20701, 1∶200 dilution; DNMT3a, sc-20703, 1∶200 dilution; DNMT3b sc-130740, 1∶100 dilution; Santa Cruz Biotechnology, USA). The specificity of antibodies had been confirmed by using Western blot analysis (data not shown). In the next day, the sections were washed five times with 0.01 mol/L phosphate-buffered saline (PBS; pH 7.4) for 15 min and then incubated with a biotinylated secondary antibody for 30 min at 37°C in the dark. After that, the sections were incubated with a streptavidin horseradish peroxidase solution for another 30 min (LSAB kit; Dako, Glostrup, Denmark), washed in PBS, and stained with DAB (3, 3-diaminobenzidine). Finally, the sections were counterstained with Mayer’s hematoxylin, dehydrated, and mounted. Negative controls were run in parallel, and were generated by PBS replacing the anti-DNMTs antibody.

### Evaluation of Immunohistochemistry

The immunostained sections were reviewed and scored independently by two investigators who were blinded to the patients’ clinicopathological characteristics. The nuclear expressed DNMTs are the functional type of DNMT proteins; therefore, only nuclear positivity for the DNMTs proteins was evaluated using semi-quantitative scoring criteria according to the staining intensity (0, negative; 1, weak; 2, moderate; and 3, severe) and proportion of positive cells (0, negative; 1, positive in ≤10%; 2, positive in >10% and ≤50%; 3, positive in >50% and ≤80%; 4, positive in >80% of tumor cells). The two scores were multiplied for each case and the expression was graded as: negative, score = 0; weak expression, score = 1–4; moderate expression, score = 5–8; and strong expression, score = 9–12.

### Statistical Analysis

Comparison of DNMT expression scores between samples was analyzed by using the Mann-Whitney U-test. Spearman rank correlation test was performed to analyze the association between DNMT expressions. Correlations between clinicopathological factors and DNMTs expression were analyzed by using the Chi-square (X^2^) test or Fisher’s Exact Probability Test. Survival of the patients according to DNMT expressions was analyzed by using Kaplan–Meier curve analysis with the log-rank test. Cox regression analysis was used for the multivariate analysis. Statistical significance was defined as *P*<0.05. All statistical tests were carried out by using the SPSS software package (SPSS 11.5 Inc, Chicago, IL, USA).

## Supporting Information

Figure S1 **Kaplan-Meier survival analysis of association between DNMT1 expression and OS and DFS in different subgroups.**
(TIF)

Figure S2 **Kaplan-Meier survival analysis of association between DNMT3a expression and OS and DFS in different subgroups.**
(TIF)

Figure S3 **Kaplan-Meier survival analysis of association between DNMT3b expression and OS and DFS in different subgroups.**
(TIF)
