# Supplementary figures and images for: Clinicopathological Significance and Prognostic Value of DNA Methyltransferase 1, 3a, and 3b Expressions in Sporadic Epithelial Ovarian Cancer
Source: PLoS One. 2012 Jun 29;7(6):e40024. doi: 10.1371/journal.pone.0040024 (PMC3386927; doi:10.1371/journal.pone.0040024)

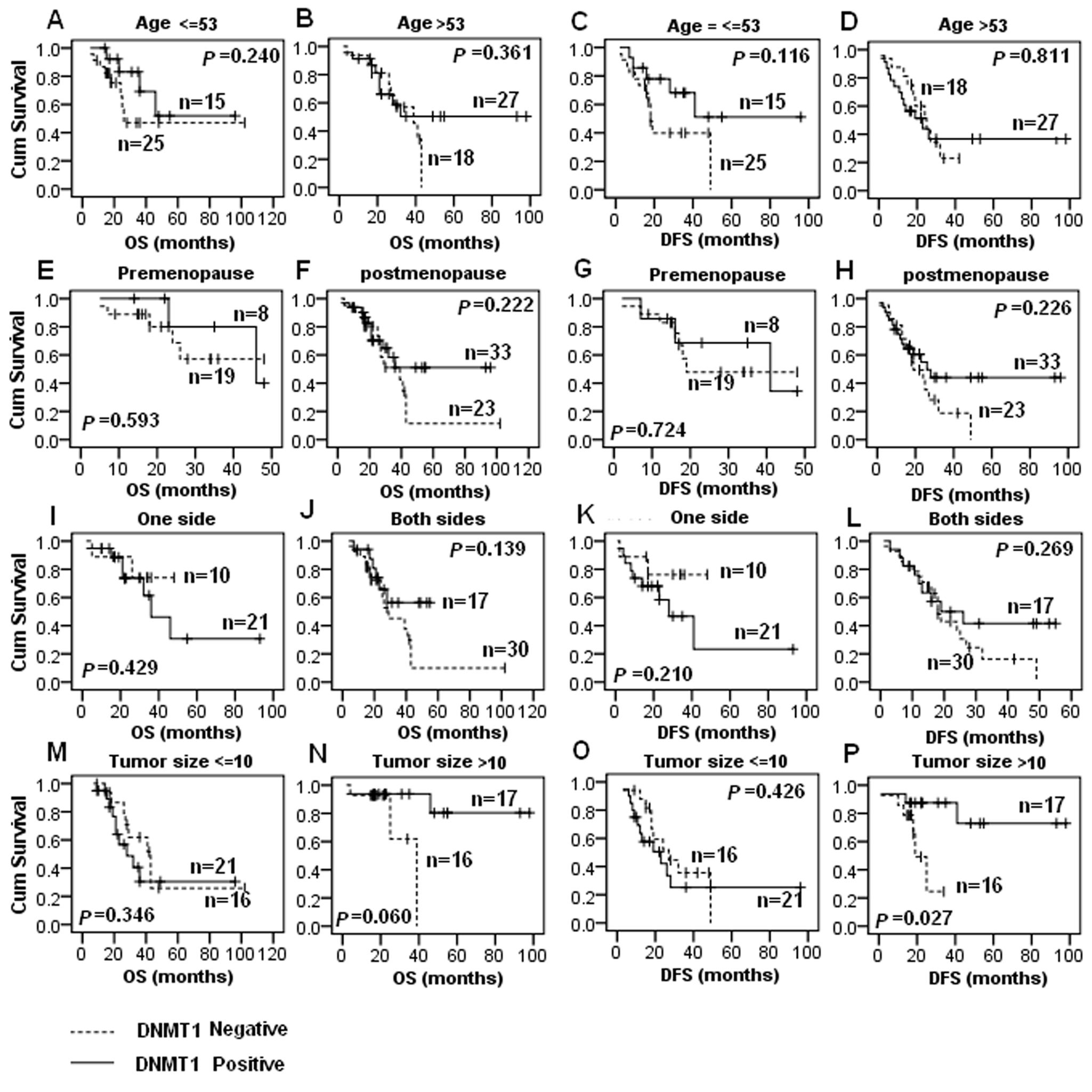

Supplement: Figure S1 — Kaplan-Meier survival analysis of association between DNMT1 expression and OS and DFS in different subgroups. (TIF) [file pone.0040024.s001.tif]

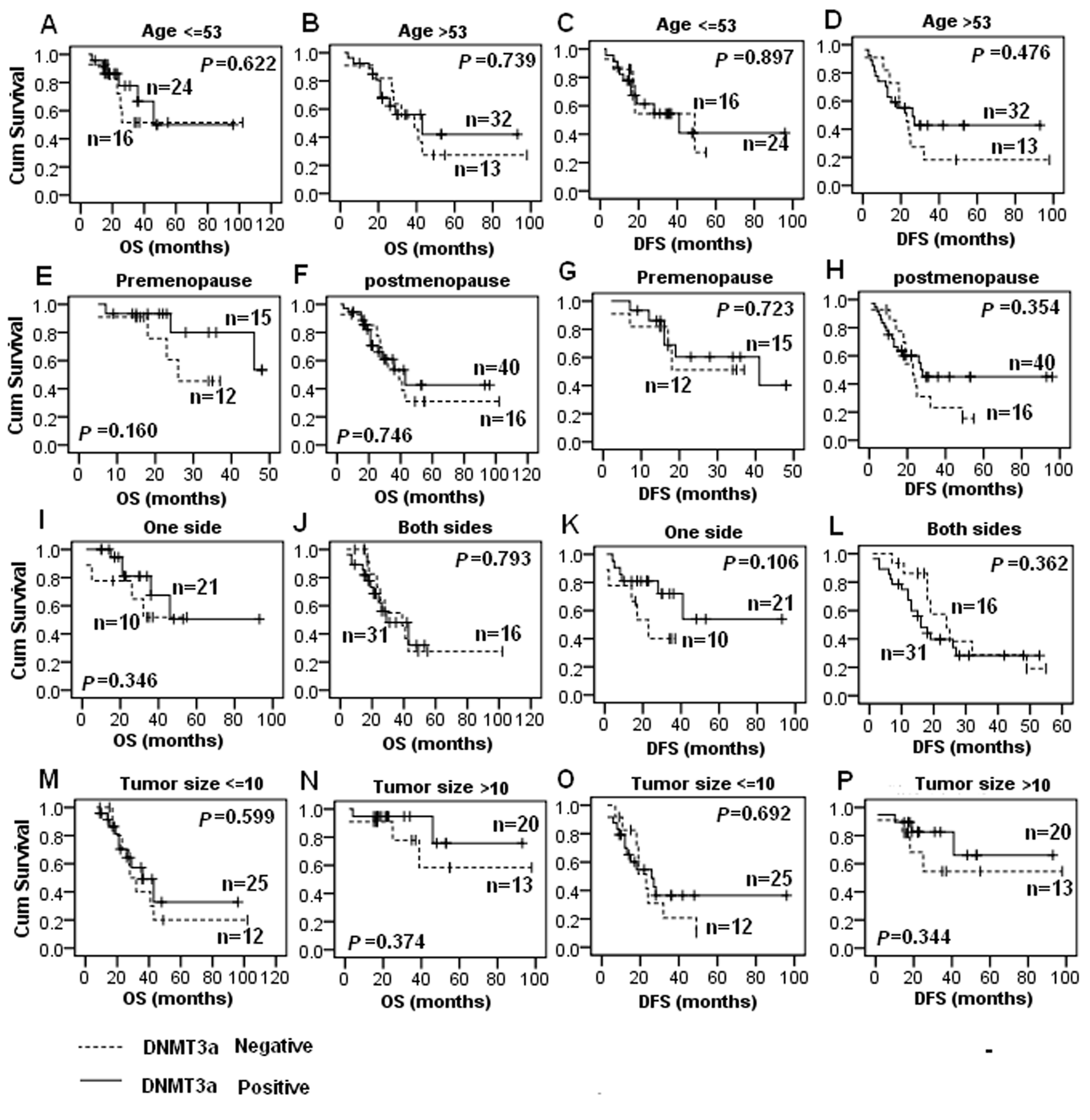

Supplement: Figure S2 — Kaplan-Meier survival analysis of association between DNMT3a expression and OS and DFS in different subgroups. (TIF) [file pone.0040024.s002.tif]

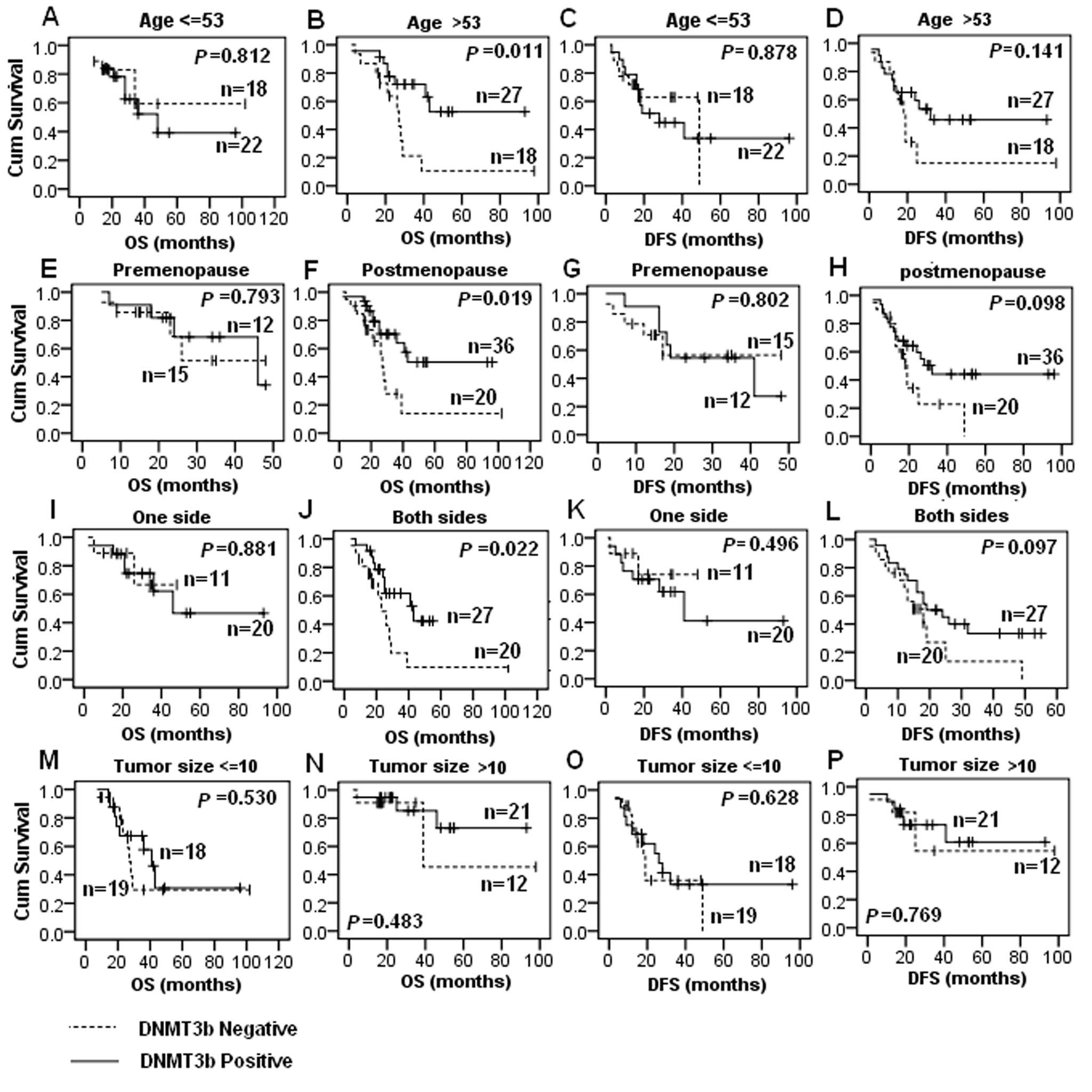

Supplement: Figure S3 — Kaplan-Meier survival analysis of association between DNMT3b expression and OS and DFS in different subgroups. (TIF) [file pone.0040024.s003.tif]
